# Unveiling the Role of Sweet Potato Root in Skin Health: A New Approach to Collagen Synthesis and Rejuvenation

**DOI:** 10.1002/fsn3.70281

**Published:** 2025-05-25

**Authors:** Ayesha Bibi, Sammra Maqsood, Muhammad Tayyab Arshad, Ali Ikram, Kodjo Théodore Gnedeka

**Affiliations:** ^1^ Department of Human Nutrition Women University Mardan Pakistan; ^2^ National Institute of Food Science and Technology University of Agriculture Faisalabad Faisalabad Pakistan; ^3^ University Institute of Food Science and Technology The University of Lahore Lahore Pakistan; ^4^ Togo Laboratory: Applied Agricultural Economics Research Team (ERE2A) University of Lomé Lomé Togo

**Keywords:** collagen, health, skin, sweet potato

## Abstract

Collagen synthesis is inextricably linked to skin health and is necessary for maintaining the skin's suppleness, structure, and general youthfulness. Wrinkles and loss of skin firmness are due to a reduction in collagen synthesis with age. The interest in dermatology lies in natural substances that promote collagen synthesis and prevent skin aging. Rich in bioactive compounds such as beta‐carotene, anthocyanins, and flavonoids, the root of sweet potato could be seen to offer the potential to enhance skin health. The review paper discusses the antioxidant and anti‐inflammatory properties of the root that protect the skin from oxidative stress and inflammation and delves into its use as a natural collagen formation and rejuvenation agent for the skin. Such in vitro and in vivo experiments and clinical studies provided experimental data on the effectiveness of this food supplement in skin health and collagen production. In addition, this review explored the role of natural sweet potato root, for instance, through the mechanism of sweet potato extract containing resveratrol in promoting skin health. This opens a chance for discussing the implications that supplements from the root of the sweet potato might make if orally administered and applied topically to improve health conditions for the skin.

## Graphical abstract

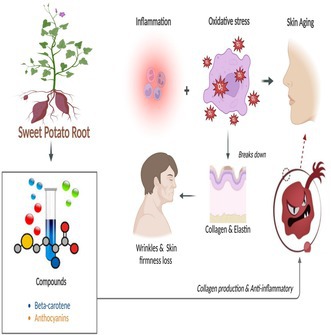

## 1 Introduction

Skin health is important for physical appearance and a defense mechanism for the body. The skin is the largest organ and protects against UV rays, bacterial infections, and other environmental factors (Kabashima et al. 2019). With age, skin function, structure, and appearance degrade, a normal condition influenced by internal and external stimuli. The most observable changes include wrinkles, reduced skin suppleness, and thinning (Reilly and Lozano 2021). Collagen is a structural protein responsible for maintaining the tensile strength and suppleness of the skin, serving as the major scaffolding component of the dermis (de Miranda et al. 2021).

The natural decline of collagen production over time mainly contributes to wrinkles and sagging skin (Al‐Atif 2022). Besides building the skin's structure, collagen helps the dermal component in wound healing and general functioning (Barati et al. 2020). Throughout the collagen synthesis process, the fibroblasts within the dermis synthesize the collagen fibers; however, this synthesis decreases as age progresses or with the effects of environmental stress (Zasada and Budzisz 2019). Therefore, collagen synthesis principles are essential to dermatologists' formulation of treatments to counteract skin aging. This involves exploring natural agents that enhance the synthesis of collagen and stimulate the renewal of the skin. Both doctors and cosmetic consultants are now anxious about maintaining the collagen levels in the skin through food or supplements (Bolke et al. 2019).

Recent studies have explored how plant‐derived antioxidants may enhance skin function via increased collagen synthesis and protection against oxidative stress (Silva‐Correa et al. 2021). Our epidermis's ability to keep moisture and softness fades as we age so producing dry, rough, and inelastic skin. The most critical aspect of cutaneous aging is the loss of collagen (Wu et al. 2024; Choi et al. 2019). Methods that protect or strengthen collagen fibers must be used to keep our skin looking young and healthy. Natural molecules have gained more attention in dermatology due to their ability to stimulate collagen synthesis, retard the aging process, and improve skin health. For example, antioxidants, vitamins, and phytochemicals in plant‐derived bioactive constituents avert oxidative stress, inflammation, and skin photoaging, thereby keeping the skin healthy (Michalak 2022). These plant‐based complexes, polyphenols, flavonoids, and anthocyanins would potentially be skin renewal mediators because they can arouse the production of collagen, protect the skin cells from ultraviolet rays, and activate the healing system within the body (Han et al. 2025; Neela and Fanta 2019; Guo et al. 2024).

Sweet potatoes ( *Ipomoea batatas* ) are among the most stimulating communal natural agents used to endorse healthy skin because of their high nutritional worth and a wide diversity of bioactive compounds (Noreen et al. 2024). The rich content of antioxidants, vitamins A and C, and anthocyanins in sweet potatoes makes them essential for producing collagen and for maintaining healthy skin (Islam 2024). Vitamin A, for example, plays a crucial role in regulating skin cell turnover and arresting collagen fiber breakdown (Reilly and Lozano 2021).

Vitamin C, a collagen synthesis cofactor, catalyzes the hydroxylation of proline and lysine, which is vital for the constancy of collagen structures (Pullar et al. 2017). Moreover, the phenolic compounds present in sweet potatoes comprise anthocyanins and chlorogenic acid, which have anti‐inflammatory and antioxidant effects that help endorse collagen production and protect skin cells from damage (Krochmal‐Marczak et al. 2021). These composites scavenge free radicals that can mutilate collagen fibers and skin cells, thus plummeting oxidative stress, a major factor in skin aging (Zague 2008).

Studies have shown that extracts from sweet potato roots, particularly those with purple flesh, have strong antioxidant properties that stimulate collagen synthesis and protect the skin from photoaging (Krochmal‐Marczak et al. 2021). Sweet potato root extract has been shown to improve the healing aptitude of wounds more than its antioxidant activity, thus illustrating its potential to stimulate collagen amalgamation. Studies have indicated that topical application of sweet potato‐derived products accelerates the healing of cutaneous wounds, probably by stimulating fibroblast activity and collagen synthesis (Silva‐Correa et al. 2021). From these results, administering sweet potato extract into diets or creams can help those intending to enhance collagen synthesis in their skins and those with skin aging. One of the natural substances that has promise in aggregate collagen synthesis and rejuvenating old skin is sweet potato root. One of the key ways it supports healthy skin is through high anthocyanin content, which is high in some varieties of sweet potatoes, such as those having the color purple flesh.

According to Krochmal‐Marczak et al. (2021), these antioxidants have been proven to significantly decrease oxidative damage in skin cells, which prevents collagen degradation and encourages dermal structure regeneration. The anti‐inflammatory properties of sweet potato extract further protect the skin from environmental agents that might otherwise lead to collagen degradation and premature aging (Krochmal‐Marczak et al. 2021). Our understanding of the potential benefits of sweet potatoes has expanded due to a better understanding of their role in collagen synthesis. Panda et al. (2011) believe that tubers of sweet potatoes possess healing potentials, perhaps by stimulating collagen amalgamation in the skin. This is highly beneficial for anyone who wants to heal from wounds on their skin or make aging skin appear younger. The extract of the root of the sweet potato can stimulate fibroblasts that produce collagen; thus, it supports the skin's basis and promotes young skin (Panda et al. 2011).

Furthermore, high amounts of vitamin A in sweet potatoes are critical in collagen formation. The vitamin regulates genes that encode proteins and skin cells and controls collagen formation to enhance skin layer regeneration and skin suppleness retention (Reilly and Lozano 2021). As vitamin A levels decrease with age, including sweet potatoes in the diet helps restore this essential mineral, enhancing collagen development. Apart from vitamin A, other essential nutrients in the sweet potato's root include manganese and zinc, which are associated with multifaceted collagen metabolism and skin health (Barati et al. 2020).

Clinical investigation incorporating the root of the sweet potato in the formulation of cosmetics has shown promising findings. For example, purple sweet potato gels greatly enhanced the healing of cutaneous wounds in mice, as Silva‐Correa et al. (2021) stated. This might be because the gels significantly enhanced collagen production at the wound site. These findings suggest that bioactive compounds present in sweet potatoes can probably repair other environmental cause‐related skin damage caused by aging and UV exposure‐related skin disorders. The sweet potato's root has immense potential as a cosmetic interference for skin transformation because it promotes collagen neogenesis and cellular regeneration (Silva‐Correa et al. 2021).

It is true that the natural ingredient, the sweet potato root, really works wonders for healthy skin and an increase in collagen. High in antioxidants, vitamins, and minerals, sweet potatoes impart integrity to the skin, avert oxidative damage, and stimulate collagen synthesis. Given the need for more and more natural and plant‐based skincare products, this use of the root of sweet potato as part of the formulation for various skin care products and supplements has proven innovative and practical enough to certify the retention of healthy and young skin.

The review attempts to identify the role of the sweet potato root in human health, with its importance being pointed out regarding skin health and secondary collagen synthesis. A comprehensive review of the sweet potato root's rich phytochemical conformation, its anti‐inflammatory and antioxidant properties, and possible pathways of collagen production points to therapeutic potential in dermatology. This investigation supports sweet potato root in topical formulations for skincare or oral supplement use.

## 2 Skin Health and Collagen Synthesis

### 2.1 Skin Structure and Function

The skin is the primary organ of the human figure and consists of three chief layers: the epidermis, dermis, and hypodermis (Khalid et al. 2020). The epidermis is the outermost layer, a defense mechanism against external stimuli. Beneath that lies the dermis, containing collagen and elastin fibers, which give it its configuration and flexibility. The hypodermis, or lowest layer, consists of fatty tissue cushioning and insulating the body (Table 1) (Yousef et al. 2017).

**TABLE 1:** Key biological factors affecting skin health, collagen synthesis, and the role of sweet potato bioactive.

Factors	Key points	Enzymes & cofactors	Factors affecting collagen	Compounds	References
Skin Layers	Epidermis, dermis, hypodermis	N/A	UV radiation, pollution	N/A	Lawton (2019); Yousef et al. (2017)
Collagen in Skin	Structural protein for elasticity	Procollagen, Tropocollagen	Aging, oxidative stress	N/A	Reilly and Lozano (2021); Benítez and Montáns (2017)
Collagen Types	Type I, II, III in skin	Collagenase, Lysyl oxidase	Smoking, diet	N/A	Avila Rodríguez et al. (2018)
Collagen Synthesis	Fibroblasts produce collagen	Vitamin C, Proline, Glycine	Inflammation, stress	N/A	Pullar et al. (2017); Khatri et al. (2021)
Skin Barrier Function	Prevents water loss, protects	Ceramides, Lipids	Harsh chemicals, dehydration	N/A	Chambers and Vukmanovic‐Stejic (2020); Kabashima et al. (2019)
Oxidative Stress & Aging	Leads to wrinkles, sagging	Antioxidants, Vitamin E	UV rays, free radicals	Anthocyanins, Carotenoids	Kimura et al. (2020); Zasada and Budzisz (2019)
Collagen Supplements	Improve elasticity & hydration	Hydrolyzed collagen	Dosage, absorption	N/A	Bolke et al. (2019); de Miranda et al. (2021)
Role of Retinoids	Stimulates collagen production	Retinoic acid	Overuse can cause irritation	N/A	Zasada and Budzisz (2019)
Vitamin C & Collagen	Essential for synthesis	Ascorbic acid	Deficiency leads to scurvy	N/A	Pullar et al. (2017)
Sweet Potato & Skin Health	Rich in antioxidants	Polyphenols, Beta‐carotene	Improves hydration & protection	Anthocyanins, Phenolics	Krochmal‐Marczak et al. (2021); Jiang et al. (2024)
Anti‐Inflammatory Properties	Reduces skin redness, irritation	Flavonoids	Chronic inflammation damages collagen	Polyphenols	Alam (2021); Hong et al. (2020)
Moisture Retention	Prevents dryness & flaking	Hyaluronic acid	Cold weather, harsh soaps	Polysaccharides	Aguirre‐Cruz et al. (2020)
Collagen Breakdown	Leads to sagging skin	Matrix metalloproteinases (MMPs)	Pollution, poor diet	N/A	Khatri et al. (2021)
Hormonal Influence	Affects skin texture & elasticity	Estrogen, Androgens	Menopause, hormonal imbalances	N/A	Irrera et al. (2017)
Wound Healing	Collagen aids in skin repair	Fibronectin, Integrins	Diabetes, poor circulation	N/A	de Albuquerque et al. (2019)
Sun Damage	Causes premature aging	DNA repair enzymes	UV exposure	Beta‐carotene	Laveriano‐Santos et al. (2022)
Antioxidant Protection	Reduces oxidative stress	Superoxide dismutase (SOD)	Pollution, poor diet	Anthocyanins	Jiang et al. (2022)
Collagen Cross‐Linking	Affects skin strength	Lysyl oxidase	Excess sugar (glycation)	N/A	Zhang et al. (2024); Czajka et al. (2018)
Diet & Collagen	Nutrients affect synthesis	Zinc, Copper	Deficiencies impair formation	Polyphenols, Flavonoids	Ooko Abong’ et al. (2020)
Sweet Potato Bioactives	Support skin rejuvenation	Anthocyanins, Phenolic acids	Enhance skin elasticity	Anthocyanins, Beta‐carotene	Shamsudin et al. (2022)

Collagen fibers preserve the mechanical integrity of the skin; in the case of the dermis, they are responsible for the resilience and strength of the skin. Collagen, a structural protein in the extracellular matrix of the dermal layer, is essential for the skin's hydration, firmness, and elasticity (Zasada and Budzisz 2019). It also accelerates the healing of wounds and is liable for the overall health and appearance of the skin (Benítez and Montáns 2017).

The physiological decline in the synthesis of collagen causes wrinkles and drooping of the skin, which are age‐related visible signs of aging and are caused by various factors (Kabashima et al. 2019). The ultimate goal of rejuvenation therapy for the skin is to preserve or enhance collagen levels (Dąbrowska et al. 2018). This is because it is through these fibers that the skin attains mechanical strength, so it does not easily deform or stretch and stays young and elastic (Lawton 2019). It is a multi‐stage process and, therefore, an intricate one—collagen production. It starts by being produced in the dermis as procollagen, a precursor molecule, by the fibroblasts. Vitamin C and other cofactors are necessary for this activity, where the procollagen undergoes hydroxylation and glycosylation to form mature collagen fibers (Reilly and Lozano 2021).

**FIGURE 1:**
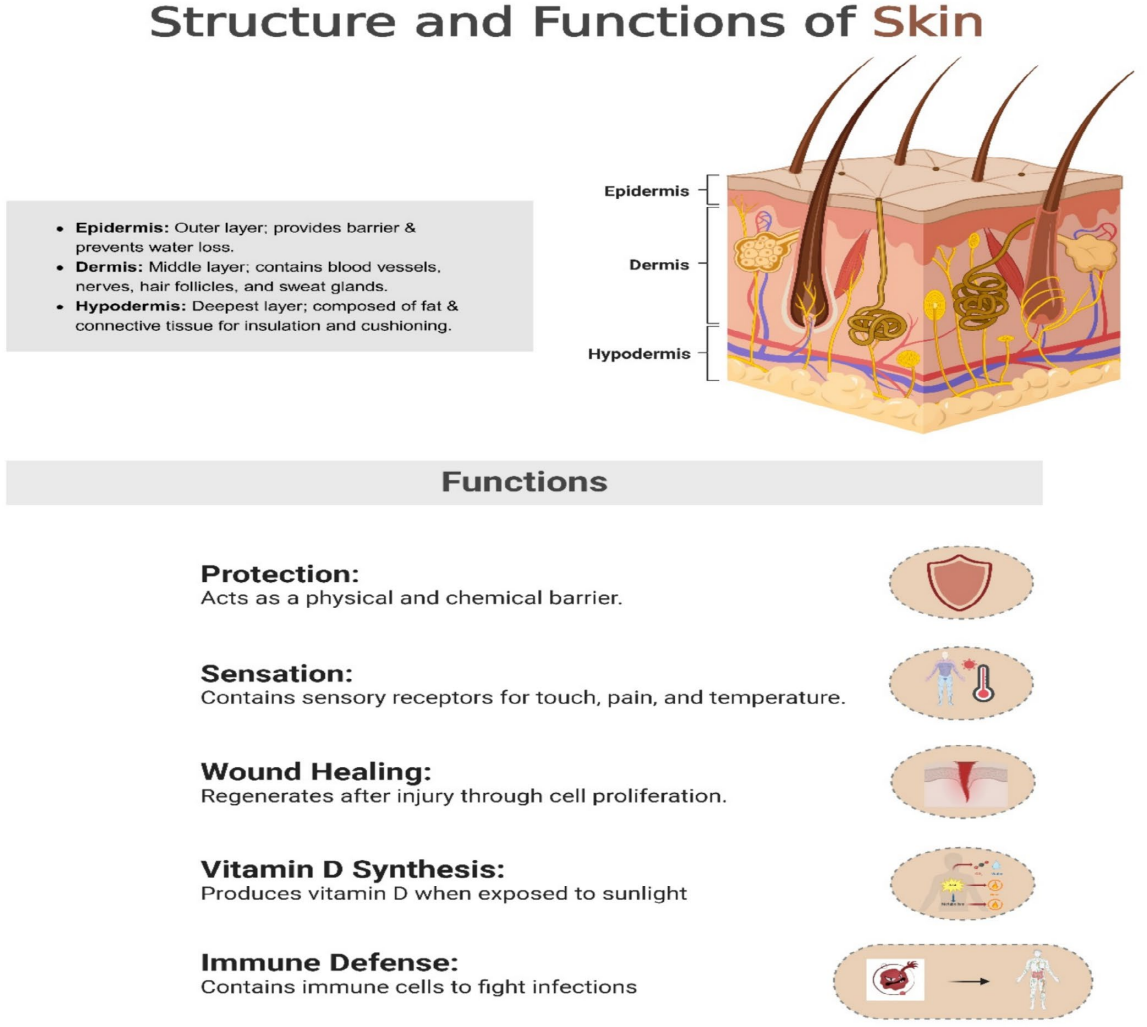
Structure and functions of skin.

Subsequently, the latter fibers align in the shape of a triple helix. With oxidative stress, aging, and environmental influences such as UV exposure, collagenase enzymes can degrade collagen faster (Figure 1) (Chambers and Vukmanovic‐Stejic 2020). Many compounds have been found through research that intend to reduce the rate of collagen degradation or increase its production to ensure topical or dietary therapy in maintaining collagen stages (Pullar et al. 2017). Examples of known treatments that either stimulate the production of new collagen or inhibit enzymes activating processes that cause degradation include collagen supplements and topical application of certain plant extracts, which show favorable therapeutic properties on skin health (Bolke et al. 2019). The findings indicate that a healthy turnover of collagen would be required to keep the skin supple and prevent or delay the appearance of aging. Table 1 depicts the key biological factors affecting skin health, collagen synthesis, and the role of sweet potato bioactive.

### 2.2 Collagen Synthesis and Its Regulation

The body contains a complex metabolic pathway for producing collagen, the most abundant structural protein. The fibroblasts start the process by making the procollagen precursor. Two significant enzymes in the aggregation of collagen are the hydroxylase enzymes prolyl hydroxylase and lysyl hydroxylase; these essentially catalyze the hydroxylation of proline and lysine residues, correspondingly. The constancy of the triple helix of collagen is based on this modification. Additionally, it requires cofactors such as vitamin C, which enables the processes of enzymatic hydroxylation for collagen synthesis (Yousef et al. 2017; Zasada and Budzisz 2019). Since it encourages the activities of prolyl hydroxylase and lysyl hydroxylase, vitamin C is necessary for the synthesis of collagen. When proline and lysine remain unhydroxylated improperly because of a deficiency of vitamin C, the strength of the collagen fibers weakens (Zasada and Budzisz 2019).

Besides, the development of the collagen matrix is further supported by minerals such as copper because these elements encourage lysyl oxidase. This enzyme cross‐links collagen fibers and alleviates the extracellular matrix (Reilly and Lozano 2021). As the body ages, it starts synthesizing less collagen and degrading the existing one. Some of the causes for this imbalance are hormonal fluctuations, oxidative stress, and the loss of the ability of the skin to heal the damage. Oxidative stress from free radicals accelerates collagen deterioration, as free radicals are produced through various environmental factors, such as pollution, smoking, and UV exposure. Reactive oxygen species activate matrix metalloproteinases to break down collagen and other extracellular matrix components (Kabashima et al. 2019; Chambers and Vukmanovic‐Stejic 2020).

Another factor is UV light from sunlight, which causes damage by breaking collagen fibers, as stated by Dąbrowska et al. (2018). Hence, the breaking up of fibers accelerates wrinkle formation and lowers the skin's supple feel. Besides, environmental pollution increases oxidative stress, promoting collagen degradation, as cited in Al‐Atif (2022). These factors hasten the skin's aging process, reducing collagen density and skin laxity (Kimura et al. 2020).

**FIGURE 2:**
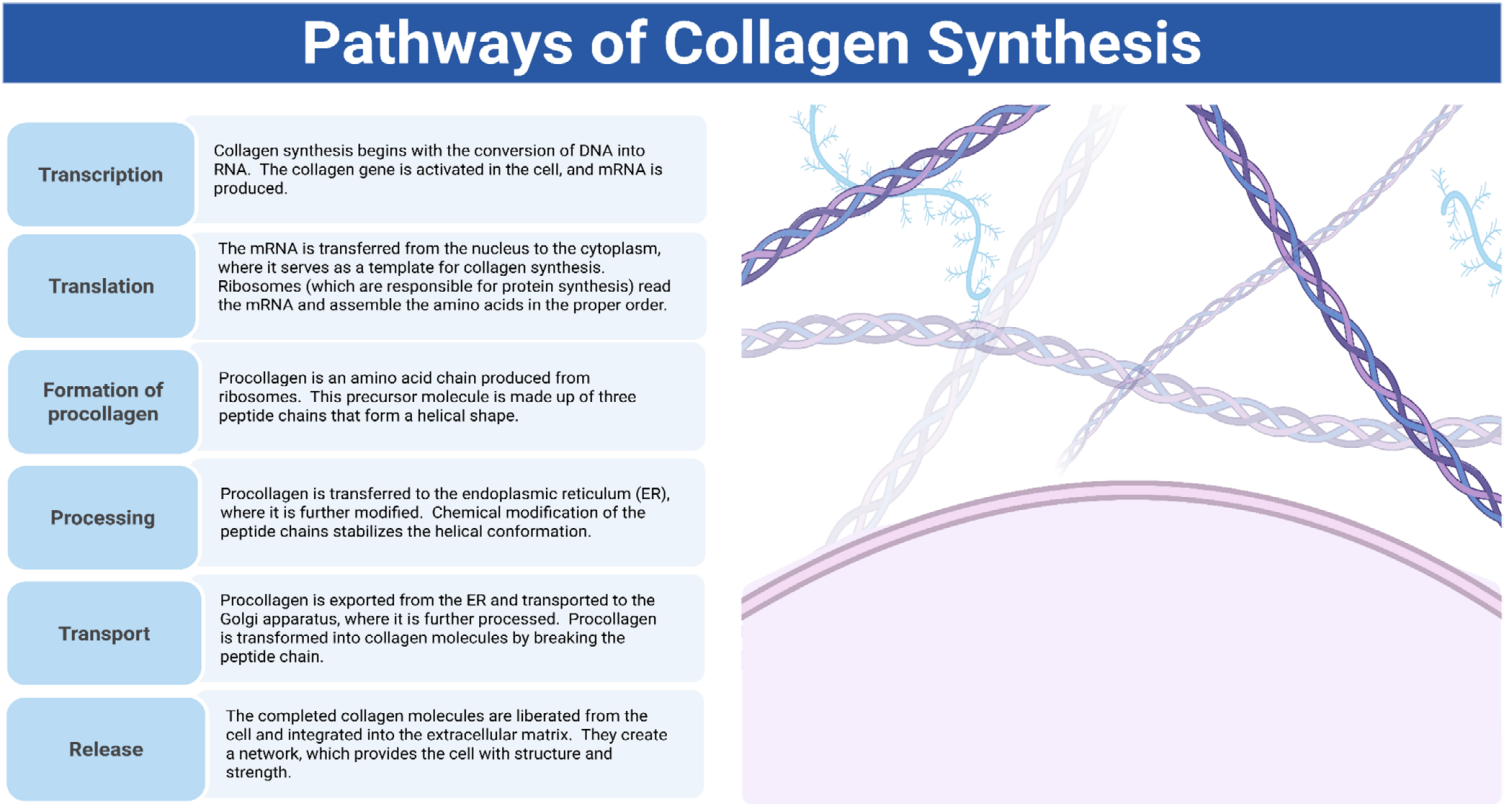
Pathways of collagen synthesis.

The most significant enzymes and cofactors in the process of the susceptible formation of collagen are prolyl hydroxylase, lysyl hydroxylase, and vitamin C. Practically, oxidative stress, aging, and environmental factors such as pollution and UV rays are the leading causes of collagen deterioration. Collagen integrity is thus critical to the skin's health and in anti‐aging methods since it is essential for protecting the structure and function of the skin. According to the new investigations, this can be associated with antioxidant or collagen peptide supplementation consistently in avoiding collagen breakage and sustaining skin health (Figure 2) (Benítez and Montáns 2017; de Miranda et al. 2021).

## 3 Sweet Potato Root

### 3.1 Phytochemical Profile of Sweet Potato Root

**FIGURE 3:**
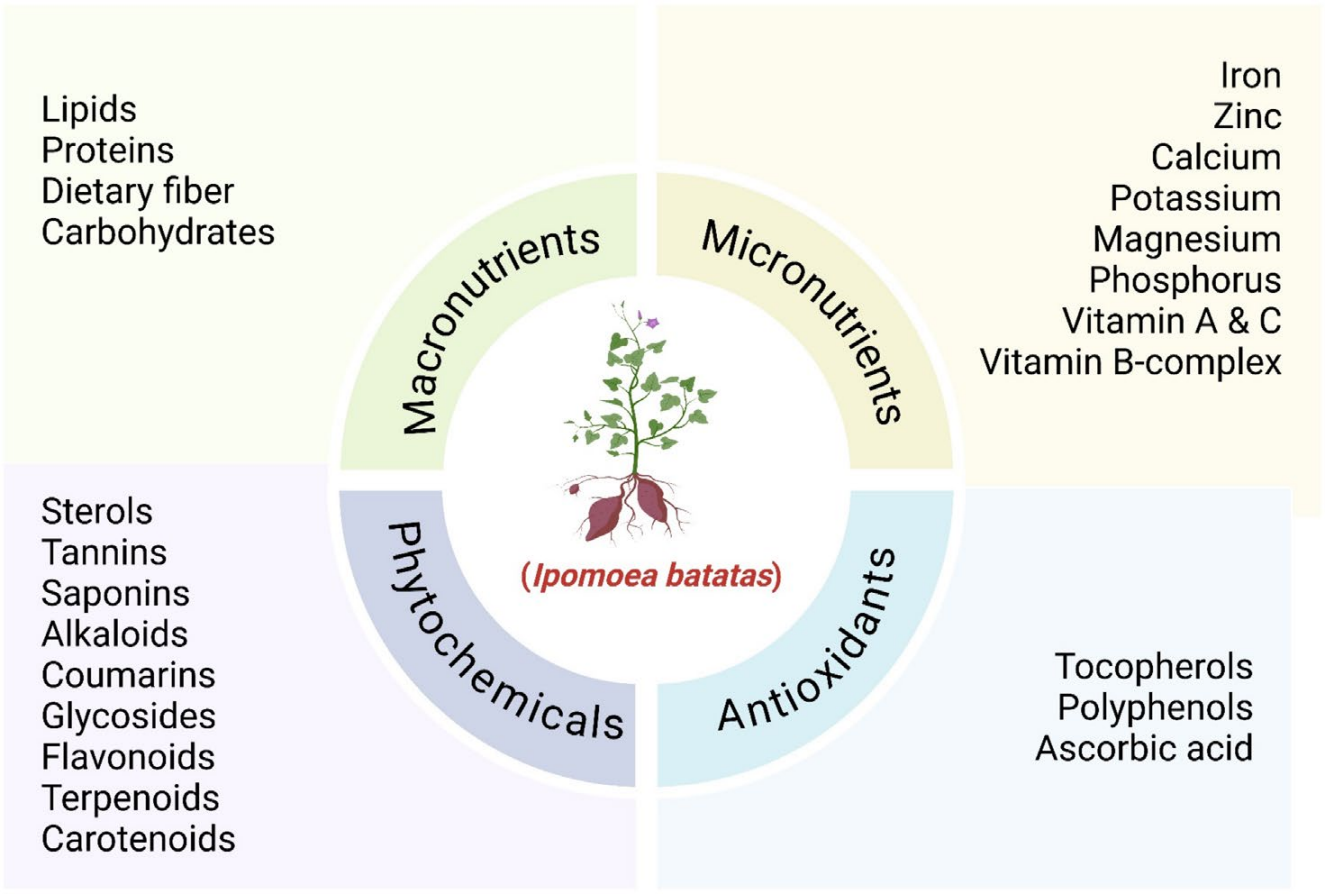
Composition of sweet potato root.

Among the several bioactive compounds present in the roots of sweet potato ( *Ipomoea batatas* ), major skin health endorsing agents are classified as beta‐carotene, anthocyanins, and flavonoids (Figure 3). Due to its function to regenerate cells and protect the skin from being damaged through light exposure, the importance of beta‐carotene, one precursor to vitamin A, is integral in preserving the integrity of the skin (Jiang et al. 2024).

Sweet potato root phytochemicals are extremely diverse, with the most bioactive ones including phenolic acids, flavonoids, anthocyanins, and chlorogenic acid derivatives (CQAs). For instance, Sun et al. (2019) reported varying levels of chlorogenic acid from 0.51 to 20.11 mg/100 g FW, as well as ferulic acid levels extending from 1.31 to 27.97 mg/100 g FW in different cultivars. Zhao et al. (2024) have recognized that purple‐fleshed types might have as much as 2003 mg/kg dry weight (DW) of anthocyanins, where cyanidin and peonidin derivatives contribute the most to this antioxidant activity. In accumulation, sweet potatoes are rich in hydroxycinnamic acids, such as 3,5‐diCQA, with anti‐inflammatory and free radical scavenging activity. These also comprise flavonoids, including hyperoside, which range from 471.3 mg/100 g FW (Sun et al. 2019; Zhao et al. 2024). Zhao et al. (2024) specify that the nutraceutical value of these foods is supplemented by the occurrence of resistant starch (0.254–9.12 g/100 g DW) and dietary fiber (7.99–26.0 g/100 g DW), which contribute to the upkeep of gut health and glycemic control. Sweet potatoes are a functional food that has the capability to alleviate the manifestations of chronic ailments because of their diverse phytochemical composition.

Orange‐fleshed sweet potatoes especially have increased interest due to their high beta‐carotene levels, which help improve skin elasticity and decrease dryness (Krochmal‐Marczak et al. 2021). Anthocyanins, the most abundant compounds in purple sweet potatoes, possess potent antioxidant and anti‐inflammatory properties that protect the skin from environmental stressors, such as pollution and ultraviolet (UV) radiation, that cause oxidative damage (Table 2) (Jiang et al. 2022). They also have been reported to stop collagen breakdown by blocking matrix metalloproteinases, which are in charge of this process (Zhi et al. 2020).

**TABLE 2:** Phytochemical profile of sweet potato root and its effects on skin health.

Bioactive compound	Category	Skin health benefit	Mechanism of action	Relevant case studies	Citation
Beta‐Carotene	Carotenoid	Enhances skin elasticity, protects against UV damage	Converted to Vitamin A, promotes cell regeneration	Protective effects on skin photodamage	Zhi et al. (2020)
Anthocyanins	Flavonoid	Reduces oxidative stress, anti‐aging	Scavenges free radicals, reduces inflammation	Antioxidant and anti‐photoaging effects	Jiang et al. (2020)
Flavonoids	Polyphenol	Anti‐inflammatory, protects against hyperpigmentation	Inhibits melanin production, modulates NF‐κB pathway	Anti‐inflammatory and skin‐protective effects	Cho et al. (2021)
Vitamin C	Antioxidant	Boosts collagen synthesis, reduces wrinkles	Enhances fibroblast function, protects against oxidative damage	Improves skin elasticity and collagen production	Pullar et al. (2017)
Polysaccharides	Complex carbohydrate	Moisturizes skin, supports wound healing	Retains water, promotes fibroblast proliferation	Enhances skin hydration and barrier function	Sun, Gou, et al. (2020)
Phenolic Acids	Antioxidant	Protects against UV‐induced damage	Reduces oxidative stress, inhibits MMP enzymes	Photoprotective effects in UV‐damaged skin	Hong et al. (2022)
Alkaloids	Bioactive compound	Enhances wound healing	Stimulates keratinocyte migration and proliferation	Promotes epidermal regeneration	Silva‐Correa et al. (2021)
Collagen Peptides	Protein derivative	Improves skin elasticity, reduces wrinkles	Stimulates collagen biosynthesis	Increases collagen density and skin hydration	Czajka et al. (2018)
Genistein	Isoflavone	Anti‐aging, antioxidant	Modulates estrogen receptors, reduces oxidative damage	Reduces skin aging markers	Irrera et al. (2017)
Vitamin E	Antioxidant	Protects against UV‐induced skin damage	Neutralizes free radicals, stabilizes skin barrier	Enhances skin barrier and hydration	Alamgir and Alamgir (2018)
Tannins	Polyphenol	Reduces skin inflammation and acne	Inhibits bacterial growth, controls sebum production	Anti‐inflammatory effects on acne‐prone skin	Sun et al. (2019)
Saponins	Glycoside	Hydrates skin, reduces irritation	Forms protective biofilm, enhances wound healing	Improves wound healing and hydration	Jiang et al. (2022)
Resveratrol	Polyphenol	Protects against oxidative stress	Inhibits ROS formation, reduces inflammation	Anti‐aging and photoprotection	Alam (2021)
Lycopene	Carotenoid	Prevents skin pigmentation, reduces sunburn	Inhibits tyrosinase activity, neutralizes UV‐induced ROS	Improves skin tone and elasticity	Laveriano‐Santos et al. (2022)
Caffeic Acid	Phenolic Acid	Prevents premature aging, protects from UV	Suppresses inflammatory mediators, inhibits MMP enzymes	Antioxidant and anti‐inflammatory benefits	Hong et al. (2022)
Ferulic Acid	Phenolic Acid	Enhances sunscreen effectiveness, reduces age spots	Stabilizes Vitamin C and E, inhibits melanin formation	Anti‐photoaging and pigmentation reduction	Jiang et al. (2024)
Proanthocyanidins	Polyphenol	Improves skin hydration, reduces wrinkles	Enhances collagen cross‐linking, reduces inflammation	Improves skin elasticity	Zengin et al. (2017)
Niacinamide	Vitamin B3	Reduces redness, hyperpigmentation	Inhibits melanosome transfer, strengthens epidermal barrier	Brightens skin and reduces signs of aging	Hoang et al. (2021)
Zinc	Mineral	Supports wound healing, reduces acne	Modulates immune response, regulates sebum production	Accelerates skin recovery	Vollmer et al. (2018)
Curcuminoids	Polyphenol	Anti‐inflammatory, protects against oxidative stress	Suppresses NF‐κB pathway, inhibits pro‐inflammatory cytokines	Reduces inflammation and enhances skin repair	Kumar et al. (2025)

The flavonoids present in the extracts of the sweet potato root have substantial free radical scavenging properties, further promoting skin health and preventing the early onset of aging (Ngcobo et al. 2024). Table 2 depicts the phytochemical profile of sweet potato root and its effects on skin health.

### 3.2 Antioxidant and Anti‐Inflammatory Properties

Inflammation and oxidative stress are the two leading causes of skin aging, which sweet potato root drastically minimizes. Oxidative stress breaks down collagen and elastin, leading to wrinkles and skin firmness loss (Alam 2021). Such significant antioxidant activity in the sweet potato root is manifested through a higher concentration of phenolic acids and flavonoids, which scavenge reactive oxygen species and control cellular damage caused by such effects (Hong et al. 2020).

Sweet potato extracts have been scientifically proven to decrease lipid peroxidation in skin cells, further aiding cellular antioxidant defense mechanisms (Krochmal‐Marczak et al. 2021). Moreover, sweet potato root has powerful anti‐inflammatory properties that decrease irritation and conditions caused by inflammation, such as dermatitis and acne (Jiang et al. 2020). It has been shown that polysaccharides isolated from purple sweet potato roots inhibit skin inflammation by inhibiting the production of pro‐inflammatory cytokines, including TNF‐α and IL‐6 (Sun, Gou, et al. 2020). It has also been validated that anthocyanins devoted to the protein in sweet potato roots may conquer the activation of the key signaling pathway linked with inflammation and skin aging, NF‐κB, according to the analysis made by Jiang et al. (2020).

Generally, sweet potato roots are favorable for collagen production and possess anti‐inflammatory and antioxidant features. Beta‐carotene and anthocyanins enhance fibroblast activity by improving the skin's architecture and collagen fabrication (Silva‐Correa et al. 2021). It has been established that topical extracts from sweet potato root improve the skin barrier function and speed up wound healing (Laveriano‐Santos et al. 2022).

**FIGURE 4:**
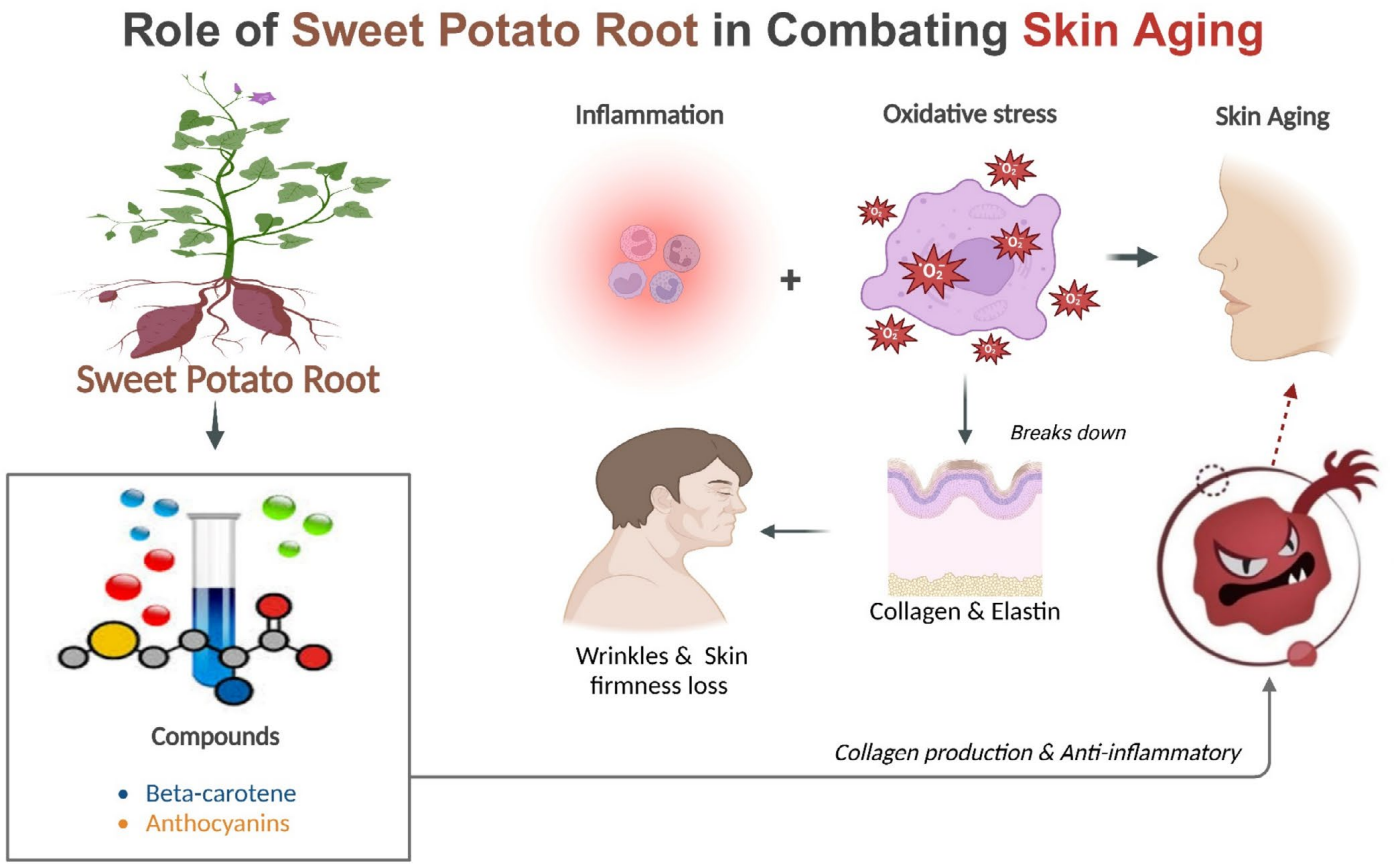
Role of sweet potato root in combating skin aging.

Due to its rich presence of phytochemicals and anti‐inflammatory and antioxidant properties, it is believed that sweet potato root is a natural ingredient that favors collagen production and, by extension, improves skin health. Future research studies should consider exploring the clinical applications of extracts produced from sweet potato roots in dermatological and cosmetic treatment (Figure 4).

### 3.3 Mechanisms Promoting Collagen Synthesis

Bioactive compounds in the root of sweet potato ( *Ipomoea batatas* ) induce fibroblast activation and inhibit collagen‐degrading enzymes. It has been established that the polyphenols and anthocyanins from sweet potatoes can induce fibroblast proliferation to synthesize new collagen for skin regeneration purposes (Zhi et al. 2020). Enhanced amalgamation of collagen due to this activation enhances the skin's suppleness and reduces the appearance of wrinkles. Purple sweet potato anthocyanins have been stated to inhibit MMPs and collagen‐degrading enzymes (Krochmal‐Marczak et al. 2021).

Anthocyanins delay skin aging and maintain collagen integrity by plummeting MMP activity. Additionally, through the protection of fibroblasts from oxidative stress and inflammation, beta‐carotene, a major antioxidant in sweet potatoes, enhances the synthesis of procollagen (Hong et al. 2022). These processes may postulate that supplementing diet and skincare products with bioactives from sweet potatoes may help preserve the integrity of collagen and recover the ailment of the skin. Numerous in vitro and in vivo studies support the role of sweet potato root in the formation of collagen and skin health. In vitro, purple sweet potato extracts rich in anthocyanins have been established to dramatically increase collagen type I expression in human dermal fibroblast cultures (Zhi et al. 2020).

The results suggest that the bioactives of sweet potatoes also decrease the inflammatory markers associated with skin aging and protect fibroblasts from oxidative impairment (Hong et al. 2022). These findings are further corroborated by in vivo studies. Anthocyanin from purple sweet potato supplementation decreased collagen degradation and enhanced the hydration of rat skin damaged by UV (Zhi et al. 2020). Moreover, the treatment of hypertensive rat models in an experimental study that used an amalgamation of purple sweet potato extract and ramipril decreased cardiac collagen degradation, indicating its potential for being used as a medicine that prevents the loss of collagen (Bhuana et al. 2020).

The effects of chemicals derived from sweet potatoes on the skin are also being studied in human clinical trials. In one study, participants took orally a sweet potato extract for 12 weeks, significantly increasing moisture, suppleness, and overall appearance of the skin in their bodies (Garner et al. 2017). A related study further designated the potential use of purple sweet potato gel for dermatological applications by showing enhanced collagen deposition and faster skin regeneration upon topical administration of the gel on mice (Silva‐Correa et al. 2021).

## 4 Nutritional and Topical Applications of Sweet Potato Root

Sweet potato root could be applied as a topical skin and orally supplemented to provoke collagen synthesis and healthy skin. Sweet potatoes contain a rich supply of vitamin C, beta‐carotene, and anthocyanins required to produce collagen (de los Ángeles Rosell et al. 2024). Beta‐carotene provides photoprotection and prevents oxidative stress‐induced collagen deprivation, and vitamin C is essential for hydroxylation, a step required for collagen constancy (Balić and Mokos 2019).

Regular consumption of sweet potatoes, either in the form of whole food or supplement, could enhance skin elasticity and retard aging. Besides nutritional uptake, chemicals isolated from sweet potatoes are increasingly valuable for topical cosmetic products. Scientific investigation finds that anthocyanins and polyphenols from sweet potatoes possess potent anti‐inflammatory and antioxidant properties, so they are perfect additives for any anti‐aging serum or cream (Hoang et al. 2021). These substances decrease inflammation and combat free radicals while refining skin texture. Additionally, encapsulation techniques have been studied to augment the stability and bioavailability of sweet potato extracts in cosmetic formulations (Ribeiro et al. 2021).

The present advancement in green extraction techniques has discovered the effective integration of sweet potato phytochemicals into cosmeceuticals (Valisakkagari et al. 2024). Skincare products have also indicated an increase in sweet potato extracts in hydration, a reduction in the hyperpigmentation of the skin, and promoting wound healing (Silva‐Correa et al. 2021).

**FIGURE 5:**
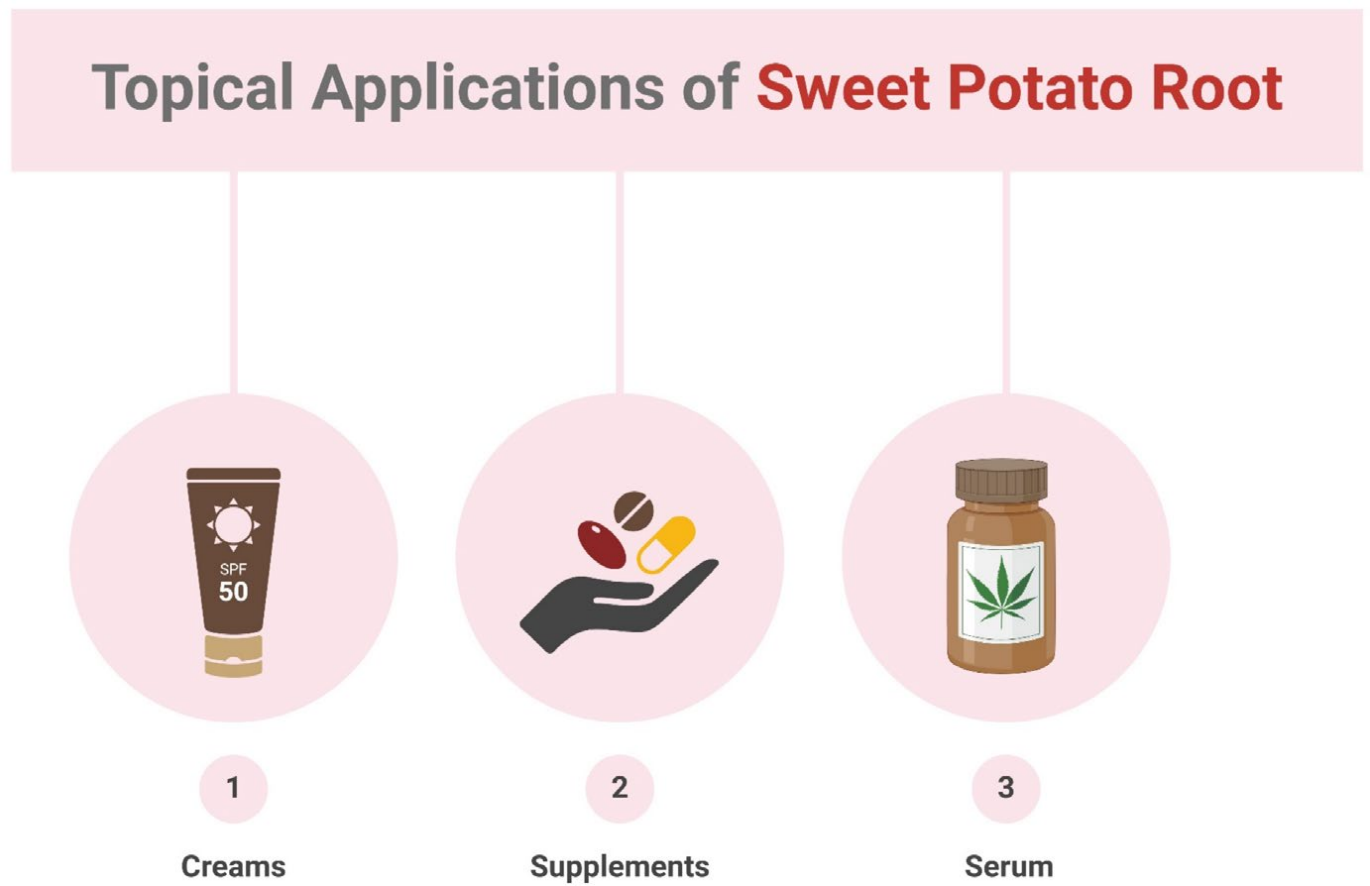
Topical applications of sweet potato root.

Oral supplements and topical treatment create a complete approach toward stimulating collagen synthesis and improvement of the skin. Sweet potato root holds promise as a natural preservative of skin health through fibroblast activation, collagen, and antioxidant protection. Not only has it been proven in both in vitro and in vivo studies to increase collagen formation and reduce oxidative stress‐induced skin aging, but it may also be approached practically by adding sweet potato bioactives to topical preparations or dietary supplements. Better formulations and the potential long‐term consequences of chemicals prepared from sweet potatoes on the skin would be established if more clinical studies were conducted (Figure 5).

## 5 Synergistic Effects of Different Bioactive Compounds in Sweet Potato Root on Skin Health

Plant‐based bioactive chemicals have garnered significant interest for their role in skin health, particularly when blended with substances that can possess anti‐inflammatory and antioxidant properties. Sweet potato root, a potential natural ingredient, is rich in beta‐carotene and other phytonutrients. The combined effect of resveratrol from grape extract with other potent antioxidants might synergize to strengthen skin protection, collagen synthesis, and general vitality. This study investigates how sweet potato root, its bioactive compounds, and their interaction with other antioxidants influence skin health. Beta‐carotene, a precursor of vitamin A, is found in the sweet potato root for healthy skin. Beta‐carotene also promotes photoprotection and lowers oxidative damage caused by UV rays. More importantly, the anthocyanins of purple‐fleshed varieties possess excellent antioxidant and anti‐inflammatory activities (He et al. 2021).

These bioactive compounds prevent premature skin aging, reduce oxidative stress, and counterbalance free radicals. Since sweet potatoes contain flavonoids and other phenolic chemicals alongside beta‐carotene, they also have flavonoids and phenolic chemicals, which exert protective effects on the skin (Rodriguez et al. 2024). These compounds modulate inflammation through their ability to reduce redness and irritation. When combined with more polyphenols, such as resveratrol, the antioxidant activity of sweet potato root can be maximized. Resveratrol induces anti‐aging and collagen‐stimulating benefits in the skin (Michalak 2022).

The amalgamation of resveratrol from grape extract and beta‐carotene from sweet potato root presents a robust synergy in skin health. Resveratrol has been proven to activate sirtuins, proteins in cellular repair and lifespan (Khmaladze et al. 2020). This mixture would bolster the skin's natural defense against oxidative stress and inflammation with beta‐carotene as part of this blend. In Chen et al. study released in 2022, synergies occur with mixes of multiple phytochemical dietary nutrients. Further, antioxidants can amplify their protecting capacities when given together. Eventually, the current blend of sweet potato root and resveratrol may enhance hydration in the skin, decrease wrinkles and fine lines, and stimulate the fabrication of collagen (Chen et al. 2022). It has also been shown that both compounds have beneficial effects on the skin microbiota, promoting a healthy and stable skin barrier (Hoang et al. 2021).

The high antioxidant action of the sweet potato root is attributed primarily to its rich carotenoid, anthocyanin, and polyphenol content. Through scavenging ROS, these compounds protect skin cells from oxidative damage (Meléndez‐Martínez et al. 2019). In return, they lower inflammatory cytokines responsible for acne, dermatitis, and psoriasis. The anti‐inflammatory property further heals the skin (Zhu et al. 2025) Mounika et al. (2024). Beta‐carotene plays a vital role in collagen synthesis by maintaining dermal fibroblast movement and increasing the skin's elasticity (Table 3) (Muzumdar and Ferenczi 2021).

**TABLE 3:** Synergistic effects of sweet potato root on skin health.

Key aspect	Bioactive compounds	Mechanisms of action	Synergistic effects	Potential benefits	Supporting studies	Applications
Antioxidant Activity	Beta‐carotene, anthocyanins, flavonoids	Neutralizes free radicals, reduces oxidative stress	Enhances skin barrier, reduces wrinkles	Anti‐aging, UV protection	Hoang et al. (2021), He et al. (2021)	Skincare formulations
Inflammation Reduction	Polyphenols, flavonoids, carotenoids	Inhibits inflammatory pathways, reduces cytokine production	Works with resveratrol to soothe skin	Reduced redness, irritation	Alam (2021), Rodriguez et al. (2024)	Anti‐inflammatory creams
Collagen Synthesis	Vitamin C, anthocyanins	Stimulates fibroblasts, enhances collagen production	Strengthens skin elasticity	Anti‐wrinkle, firming effects	Mounika et al. (2024), Chen et al. (2022)	Anti‐aging serums
UV Protection	Phytoene, phytofluene, beta‐carotene	Absorbs UV radiation, reduces photodamage	Synergizes with other carotenoids	Prevents pigmentation, sunburn	Meléndez‐Martínez et al. (2019), Oliveira et al. (2020)	Sunscreens, UV‐blocking lotions
Moisture Retention	Polysaccharides, ceramides	Enhances epidermal barrier, locks in moisture	Works with lipid‐based nutrients	Hydrated, supple skin	Tessema et al. (2017), Kyriakoudi et al. (2021)	Moisturizers, hydrating masks
Hyperpigmentation Reduction	Anthocyanins, flavonoids	Inhibits melanin synthesis, reduces dark spots	Works with vitamin C	Brighter, even skin tone	Islam (2024), Escobar‐Puentes et al. (2022)	Brightening serums
Wound Healing	Phenolic acids, flavonoids	Promotes cell regeneration, reduces scar formation	Synergizes with antimicrobial agents	Faster healing, reduced scarring	Xuan et al. (2024), Sultana et al. (2024)	Wound‐healing creams
Anti‐Aging Effects	Anthocyanins, polyphenols	Reduces oxidative stress, boosts skin regeneration	Works with retinoids	Smoother, youthful appearance	Villalba et al. (2024), Armadany et al. (2024)	Anti‐aging skincare
Anti‐Acne Effects	Phenolics, flavonoids	Reduces bacterial growth, controls sebum production	Complements salicylic acid	Clearer, acne‐free skin	Wan et al. (2024), Dias et al. (2021)	Acne treatments
Skin Barrier Function	Lipids, polyphenols	Strengthens skin defenses, prevents water loss	Enhances with ceramides	Resilient, less sensitive skin	Gupta et al. (2024), Bushra et al. (2024)	Barrier creams
Antimicrobial Properties	Saponins, tannins	Inhibits bacterial and fungal growth	Works with antimicrobial peptides	Prevents infections	Moura et al. (2021), Ilhan and Ozturk (2024)	Antiseptic skincare
Skin Detoxification	Anthocyanins, polyphenols	Reduces toxin buildup, enhances skin metabolism	Combines with herbal detoxifiers	Clearer, healthier skin	Xiao et al. (2023), Goyal and Chauhan (2024)	Detox masks, toners
Scar Reduction	Resveratrol, flavonoids	Promotes tissue regeneration	Synergizes with peptides	Fades scars	Meng et al. (2024), Das et al. (2019)	Scar gels, healing serums
Skin Firming	Flavonoids, peptides	Improves elasticity, prevents sagging	Works with hyaluronic acid	Tighter, youthful skin	He et al. (2021), Oliveira et al. (2020)	Firming serums
Cellular Repair	Antioxidants, amino acids	Enhances DNA repair, reduces oxidative damage	Supports cellular turnover	Healthier, radiant skin	Sakamoto and Suzuki (2024), Li et al. (2021)	Regenerative skincare
Hydration Enhancement	Starches, polysaccharides	Forms protective film, locks in moisture	Boosts humectant effects	Long‐lasting hydration	Yuan et al. (2022), Marto et al. (2017)	Hydrating serums
Anti‐Glycation Effects	Phenolics, flavonoids	Prevents sugar‐induced skin damage	Synergizes with vitamin E	Delays skin aging	Tedesco et al. (2023), Sivamaruthi et al. (2022)	Anti‐aging creams
Exfoliation Support	Enzymes, organic acids	Gently removes dead skin cells	Enhances fruit acid exfoliators	Smoother skin texture	Islam (2024), Ashfaq et al. (2023)	Peels, exfoliating masks
Nutrient Absorption	Bioactive compounds	Improves skin's uptake of nutrients	Works with liposomal formulations	Maximized skincare benefits	Kyriakoudi et al. (2021), Ali et al. (2019)	Delivery‐enhancing skincare

Resveratrol enhances this effect by inhibiting matrix metalloproteinases (MMPs), which degrade collagen and lead to skin aging (Ribeiro et al. 2021). Thus, combining these composites may lead to more flexible and younger skin. The plant has much potential to improve skin health since sweet potato root comprises many bioactive compounds. It can enhance antioxidant activity, reduce inflammation, and strengthen collagen amalgamation when joined with resveratrol and other robust antioxidants. By realizing the importance of introducing plant‐derived composites into skincare, this set of actions has ushered in a new and more natural way of keeping skin firm and healthy. Future research will be focused on finding the ideal dosages and combinations to further enhance the potency of these bioactives for dermatological purposes. Table 3 depicts the synergistic effects of sweet potato root on skin health.

## 6 Challenges and Future Directions

Even as the potential benefits of sweet potato roots ( *Ipomoea batatas* ) in collagen synthesis and skin health are increasingly better recognized, many questions remain (Chen et al. 2019). Most current studies are limited to in vitro or animal models, even though earlier studies pointed out sweet potato root extracts' antioxidant, anti‐inflammatory, and collagen‐inducing properties (Islam 2024; Alam 2021). Because human skin is a multi‐layered organ and research on bioactive substances may cause varied reactions, animal studies cannot always be transferred and translated to the scope of human skin health. In addition, the range of bioactive compounds found in sweet potato roots has been ignored for research focused on certain compounds such as carotenoids or anthocyanins (Nguyen et al. 2021).

The accurate mechanisms through which sweet potato root constituents influence human skin health, particularly regarding collagen synthesis and skin aging, should be further studied. Long‐term research is needed to understand the safety and efficacy of sweet potato root bioactive compounds, although short‐term research into the root's effects on the skin is promising. While considering a lack of research on the long‐term effects of sweet potatoes on skin health, mainly when applied topically as skincare or ingested through diet, the vast majority of studies conducted thus far have focused on short‐term or immediate effects, including antioxidant and anti‐inflammatory activities (Hsu and Chen 2022).

Unintended side effects may occur, such as dermatological hypersensitivity or adverse reactions to extended application or consumption of sweet potato products. These composites must be established with long‐term safety profiles to confirm their effectiveness and minimize dangers as consumers' attention to natural skincare increases (Alam 2021). Long‐term research in one of the areas can considerably contribute to assessing sweet potatoes' impact on skin aging. Short‐term experiments are insufficient to test the stimulation of long‐term collagen synthesis because the breakdown of collagen, one of the main constituents of skin aging, can be years before being manifested in humans. Thus, to validate the importance of sweet potatoes in averting or reversing ciphers of aging, longitudinal clinical experimentations and studies integrating real‐life practice over months or even years would be precious (Tessema et al. 2017). However, one major limitation of using sweet potato roots for skin health is the bioavailability of their bioactive components. Since the skin possesses poor absorption of them, most bioactive materials such as anthocyanins, carotenoids, and polyphenols that give most of the health benefits to sweet potatoes often exhibit reduced bioavailability (Sivamaruthi et al. 2022).

Nanotechnology and other advancements in delivery methods bring inspiring responses to this problem. Bioactive substances encapsulated in nanoparticles stabilize the nanoparticles, improve skin penetration, and ensure more effective delivery and release of the active ingredients for extended periods (Ali et al. 2019). For example, sweet potato‐derived chemicals may be more efficiently conveyed using liposomes and nanoemulsions already used in pharmaceutical and cosmetic products. These technologies may stabilize sensitive substances such as carotenoids and anthocyanins to remain potent even after a while (Ashfaq et al. 2023; Nallasamy and Natarajan 2022). Further, coupling advanced nanotechnology with the benefits of the plant bioactive composition of sweet potatoes may provide starch‐based nanoparticles as an environmentally friendly and non‐toxic alternative to synthetic delivery systems (Qiu et al. 2019).

The combination of natural components and advanced delivery technologies is vast enough to have great potential further to enhance sweet potato root's capability for skin health. Even though sweet potato root alone has a myriad of health benefits, the potential impact of the root on skin health might be enhanced in the presence of other organic materials. This may allow for an even more effective formulation by amplifying the synergistic effect of several bioactive chemicals related to collagen amalgamation, anti‐aging, and skin rejuvenation. For instance, incorporating phytochemicals from green tea, turmeric, or pomegranates with sweet potato bioactives can enhance the latter's antioxidant properties and thus offer excellent protection against skin damage caused by free radicals (He et al. 2022).

This adds ceramides and other molecules known to enhance the skin barrier and thus may lead to more thorough skin care, providing support for both the formation of collagen and the recovery of the skin's natural barrier function (Tessema et al. 2017). There is increased interest in using sweet potato bioactives alone or combined with other plant‐derived chemicals on photoprotection. Sweet potatoes are rich in carotenoids and may supplement other natural photoprotective substances, such as those in citrus fruits or berries, for a natural replacement for artificial sunscreens (Oliveira et al. 2020).

## 7 Conclusion

The bioactive components of sweet potato root make it an excellent natural agent for collagen induction and healthy skin. It helps to fight against inflammation and oxidative stress, two primary reasons for skin aging. Moreover, the stimulation of fibroblast activity and the inhibition of collagen‐degrading enzymes could cause an increase in the synthesis of collagen, hence acting as a potential natural ingredient to produce softness, moisture, and the overall appearance of the skin. Thus, in the aesthetic dermatology and cosmetics arena, sweet potato root may serve as a holistic approach to skin rejuvenation by being employed as a constituent for topical applications and nutritional supplementations. This means that more data from clinical trials and experimental investigations propose that sweet potato root has much potential for skincare and anti‐aging treatments. It is a natural source of chemicals that upsurge collagen, thus possibly supplementing or replacing traditional anti‐aging products. Further investigation is necessary to fully understand the mechanisms involved in its assistance and make the most of its use in skincare. However, additional bioactive constituents may make the root of sweet potato valuable in more health‐associated areas besides those it currently serves.

## Author Contributions

**Ayesha Bibi:** writing – original draft (equal). **Sammra Maqsood:** writing – review and editing (equal). **Muhammad Tayyab Arshad:** data curation (equal). **Ali Ikram:** validation (equal). **Kodjo Théodore Gnedeka:** formal analysis (equal).

## Disclosure

*Institutional Review Board Statement*: This study did not involve humans or animals.

## Consent

The authors have nothing to report.

## Conflicts of Interest

The authors declare no conflicts of interest.

## Data Availability

The data supporting this study's findings are available from the corresponding author upon reasonable request.
